# UHPLC-ESI-MS Analysis of Purified Flavonoids Fraction from Stem of* Dendrobium denneaum Paxt.* and Its Preliminary Study in Inducing Apoptosis of HepG2 Cells

**DOI:** 10.1155/2018/8936307

**Published:** 2018-04-22

**Authors:** Chunhua Zhou, Yingyi Luo, Zhouxi Lei, Gang Wei

**Affiliations:** School of Pharmaceutical Sciences, Guangzhou University of Chinese Medicine, Guangzhou, Guangdong 510006, China

## Abstract

*Dendrobium denneaum paxt.*, which has been widely used for health prevention in traditional Chinese medicine (TCM), is one of the most popular tonic herbs in China. In order to analyze its flavonoids, characterization and antitumor activity of crude extract and flavonoids rich fractions from* D. denneaum paxt.* were investigated. Flavonoids extracted from* D. denneaum paxt. *were clearly enriched in fraction II after determining the total flavonoids content; there were 15 characteristic peaks which have been detected; ultra-high performance liquid chromatography-electrospray ionization/mass spectrometry (UHPLC-ESI-MS/MS) was applied for structural elucidation of compounds. 13 characteristic peaks including flavonoid-O-glycosides and flavonoid-C-glycosides were determined or preliminarily characterized through comparing retention times and UV and MS spectra with standard compounds or documented literature. The antitumor activity of fraction II on human liver cancer cells HepG2 was investigated. MTT assay method was used to test the antiproliferation activity and to confirm the appropriate treatment concentration as well as inducing time. The morphological changes of the apoptosis cells after being induced by fraction II were observed by a Hoechst reagent and the apoptosis rate was tested by flow cytometry. The results showed that fraction II can inhibit HepG2 cells from proliferation in a dose-dependent and time-dependent manner. The apoptosis experiments indicated that fraction II can significantly induce apoptosis in HepG2 cells in a concentration over 50 *μ*g/mL for 48 h and the most effective level was 150 *μ*g/mL for 48 h.

## 1. Introduction

*Dendrobium* genus are used as traditional herbal remedies called shihu in China for a long period, which has been deemed as a valuable folk medicine with diversity medicinal activities in traditional Chinese medicine (TCM) theory, including promoting stomach fluid, nourishing yin, and clearing heat [1–4]. Modern pharmacological studies manifest that* Dendrobium* genus have various functions on human body such as antitumor, immune-regulation, antioxidant, antiplatelet aggregation, dilation of blood vessels, and hypoglycemic activity [5–8]. In the* Dendrobium* genus,* D. officinale* and* Dendrobium huoshanense *are the most popular species in traditional Chinese market. Since the 1950s, these two species were on the edge of extinction due to the tremendous demand in health preservation of the public. And since then, the herbal medicinal markets were filled with other* Dendrobium* species such as* D. denneaum paxt. *which accounts for the vast majority named “Zipi Shihu.” Traditionally the stems of* D. denneaum paxt. *will be heated in adequate temperature to be softened and then twisted into a spiral like a spring and dried before selling in the market [9]. After these processes the products are commonly known as Zipi Fengdou. As time goes by,* D. denneaum paxt.* has become the major source of* Dendrobium* species in traditional Chinese medicine market which occupies half of the market share. The traditional herbal medicinal practitioners have confirmed that it has similar therapeutic effect with* D. officinale*. In recent decades, the plantation area of* D. denneaum paxt.* is expanding rapidly in Longling County of Yunnan Province, China [3].

The existence and activity of free radicals are the main cause of metabolic disorder which may lead to many diseases including angiocardiopathy, diabetes, and cataracts. It is feasible to introduce antioxidant into the body for the prevention or treatment of these diseases. Flavonoids are a widespread group of phytochemistry with diverse biological functions and the significant substances in plants, which not only play a key role in the pharmaceutical industry, but also serve as the excellent chemical markers to control the quality of medicinal plants [10, 11]. Plenty of studies showed that the flavonoid glycosides have obvious pharmaceutical function of antioxidant, hypoglycemic and cardiovascular prevention activity [12–19]. Many researchers have studied the mechanism of the antioxidant and anticancer effect of flavonoids extract in plants. Thong et al. studied the mechanism of antioxidant activities of chalcone, flavones, and flavanone and found that the mechanism of antioxidant activities was activated through hydrogen atom transfer and single electron transfer mechanism [20]. Jiang et al. found that the lyophilized aqueous extracts which contained a high level of flavonoids exhibit significant antioxidant activities on mesenchymal stem cells. This kind of activities was through direct ROS-scavenging and indirect Fe2+- chelating [21]. Yuan et al. found that Isoorientin could enhance the expression level of GSH and increase phase II detoxifying enzyme activities in liver cancer cell line which may be the mechanism for Isoorientin anticancer activities [22]. Comparing to the chemical antioxidants, the natural products are healthier with fewer side effects. At present, there have been no reports on the pharmacological studies of the flavonoids from* D. denneaum paxt.* We speculated that the flavonoids in* D. denneaum paxt. *would exhibit similar activities as the flavonoids from other plant extract. Therefore, we decided to study the antitumor activity of flavonoids in* D. denneaum paxt. *by inducing HepG2 cell apoptosis.

There is a substantial content of flavonoid glycosides in the plant* D. denneaum paxt.*; it is significant for searching and screening out some strong antioxidant activities components to develop the new drug. No reports can be found in fingerprint chromatography establishment by UHPLC-MS as well as antitumor activity study of the flavonoid in* D. denneaum paxt.* so far. Therefore, it is instructive to build a method of fingerprint chromatography by UHPLC-MS and study the* in vitro* antitumor activity to evaluate the plant* D. denneaum paxt.* This study will become an experimental reference in developing a new health preserving and functional drug.

## 2. Materials and Methods

### 2.1. Chemicals and Reagents

#### 2.1.1. Chemicals and Reagents in UHPLC-MS Analysis

Apigenin-6,8-di-C-*β*-D-glucoside and Apigenin-6-C-*β*-D-xyloside-8-C-*β*-D-glucoside were isolated from the leaves of* D. officinal *by preparative liquid chromatography as reference substances for the experiments. Rutin and Schaftoside were obtained from the National Institute of the Control of Pharmaceutical and Biological Products (Guangzhou, China). The purity of all compounds mentioned above is over 96% in HPLC level and their chemical structures were identified by comparing their UV, IR, ESI/MS, and NMR data with other published literatures.

Chromatographic grade methanol was purchased from Merck (Darmstadt, Germany). Preparation of pure water was done by using A Milli-Q water purification system (MA, USA). Methanol and ammonium acetate (CH3CO2NH4) with analytical reagent grade were obtained from Damao Chemical Corporation, Tianjin, China. All standard analytical solutions were stored at 4°C.

#### 2.1.2. Materials and Chemicals in Cell Experiments

The human liver cancer HepG2 cell line was obtained from Guangzhou Jini Ou Biological Technology Co., Ltd. (Guangzhou, China). High glucose Dulbecco's modified Eagle's medium (H-DMEM), fetal bovine serum (FBS), penicillin-streptomycin, trypsin, trypsin (non-EDTA), and phosphate-buffered saline (PBS) were purchased from Gibco Chemical Co. (Rockville, MD, USA). MTT, 5-Fluorouracil, and dimethyl sulfoxide (DMSO) were acquired from Sigma-Aldrich (St. Louis, MO, USA). The Annexin V-FITC apoptosis assay kits and Hoechst 33258 assay kits were purchased by KeyGen Biotech Co., Ltd. (Jiangsu, China). The fraction II was a fraction from stem of* D. denneaum Paxt.* enriched with flavonoids.

### 2.2. Materials Sample Preparation

50 g of the stem of* D. denneaum Paxt.* was extracted by refluxing with 800 mL 70% ethanol for 1 h twice. The extraction was combined and concentrated under vacuum to 20 mL. Petroleum Ether was used to remove pigment and other impurities. Then the extraction was dried with water bath and dissolved into suspension with 30 mL of pure water. The BuOH-soluble fraction was chromatographed on AB-8 macroporous adsorption resin eluting with ethanol in different concentrations of 10%, 30%, 50%, and 90%, respectively. The four elution parts named fractions I, II, III, and IV were collected and weighed after being dried. The weights of fractions (I–IV) were 599.23 mg, 345.38 mg, 155.13 mg, and 153.64 mg. The four fractions were dissolved in HPLC grade methanol to test flavonoids.

### 2.3. UHPLC Instrumentation and Chromatographic Conditions

The UHPLC system consisting of a vacuum degasser, quaternary pump, autosampler, and ultraviolet detector (Thermo Separation Products Inc., Riviera Beach FL, USA) was used for acquiring chromatograms. The studies were carried out on a Hypersil GOLD C18 (100 × 2.1 mm ID, 1.9 *μ*ml, Thermo, USA). The mobile phase consisted of methanol (A) and 10 mM (v/v) ammonium acetate aqueous solution (B). Gradient elution at a flow rate of 200 *μ*L·min^−1^ was applied to establish fingerprint chromatography. The suitable methanol gradient was performed at the following procedure: 0–5 min, 16–20% B; 5–13 B, 20–23% B; 13–19 B, 23-24% B; 19–25 min, 24–26% B; 26–31 min, 26–28% B; 31–45 min, 28–35% B; 45–55 min, 45–55% B. The column temperature was 30°C in the whole analysis process. Sample introducing amounts were 3 uL.

### 2.4. MS Instrumentation Conditions

The MS analysis was performed on LCQ ion trap instrument (Thermo Finnigan, San Jose, CA, USA) with a heated electrospray ionization (ESI) source coupled to the LC apparatus for mass spectrometric identification of characteristic peaks. The same conditions as described above were used. For optimum MS results, the negative ion mode for MS analyses was selected and worked under the following conditions: spray voltage 3.0 kV, capillary temperature 300°C, sheath gas flow rate at 35 (arbitrary units), auxiliary gas flow rate at 10 (arbitrary units), and split ratio 4 : 1 (v/v), respectively. The full-scan mass spectra were set in the range of *m*/*z* 100–1,000. All data were manipulated by Finnigan Xcalibur 2.0 Advanced chromatography workstation (Thermo Quest Corporation, San Jose, CA, USA).

### 2.5. Total Flavonoids Content (TFC)

The TFC was measured following a described colorimetric method [23]. Rutin was used as standard and expressed as milligrams of Rutin equivalent per gram of sample (mg RE/g sample). The liner regression was *A* = 1.0451*C* + 0.0585  (*R*2 = 0.9996), where *A* was the absorbance and *C* was the concentration of Rutin equivalent (RE) mg/ml.

### 2.6. Analysis of Antitumor Activity

#### 2.6.1. The Choice of Flavonoids Fraction and Conducting Concentration

In the cell experiment section, we chose the flavonoids fraction which contained most of the flavanoids. The conducting concentrations (10, 25, 50, 100, and 150 *μ*g/mL) and duration time for cell viability were based on a routine practice of the antitumor in vitro experiments. And the conducting concentration and duration time for further experiments included cell morphological change and flow cytometry analysis was based on the cell viability results.

HepG2 cells were cultured at 37°C in a humidified atmosphere of 5% CO2 in H-DMEM medium supplemented with 10% FBS (v/v) and 1% penicillin-streptomycin. The control group were cultured without fraction II in serum-free medium while the positive drug group were cultured in serum-free medium with 5-Fluorouracil (5-Flu). The stock solution of flavonoids fraction II was prepared by dissolving flavonoids fraction II in DMSO with a concentration of 200 mg/mL in order to guarantee the DMSO content was less than 1% in the final fraction II solution. The cell experiments were done by diluting the stock solution with H-DMEM medium (without FBS) to different concentrations (10, 25, 50, 100, and 150 *μ*g/mL).

#### 2.6.2. Cell Viability Assay

The cell viability was estimated using MTT assays. Briefly, HepG2 cells were seeded in a 96-well plate (3000 cells/well) and cultured for 24 h using H-DMEM (with FBS). Then the culture medium was replaced by H-DMEM (without FBS) with fraction II in different concentrations (10, 25, 50, 100, and 150 *μ*g/mL) for 24 and 48 hours, respectively. The control group and positive drug group were under the same conditions. After the treatment, 20 *μ*l MTT (5 mg/mL) was added to each well and sequentially incubated for an additional 4 h at 37°C. The cell supernatant was removed and 100 *μ*L DMSO was added to dissolve the dark blue formazan. The absorbance was measured at 490 nm using a microplate reader (Bio-Rad Laboratories, Inc., Hercules, CA, USA) after the plate was shaken for 5 minutes. The cell viability was calculated as follows:

(1) Cell  viability=A490  experimental/A490  control∗100%.

#### 2.6.3. Cell Morphological Changes

The morphological changes of HepG2 cells after being induced by fraction II were evaluated by staining with the chromatin dye Hoechst 33258. The HepG2 cells were cultured in 6-well plates in the concentration of 1 × 105 cells/mL for 24 h and then exposed to various concentrations of fraction II (0, 50, and 150 *μ*g/mL) and 5-Flu (50 *μ*g/mL) for 48 h under the same condition. Subsequently, the cells were immobilized with 4% paraformaldehyde for 10 min at 4°C and then stained with Hoechst 33258 for 10 min in the dark after being washed twice with PBS. Morphological changes and the images of the apoptotic cells were observed using a fluorescence microscope (Leica, Microsystems GmbH, Wetzlar, Germany).

#### 2.6.4. Flow Cytometry Analysis of Cell Apoptosis

HepG2 was seeded in 6-well plates (1 × 105 cells/mL) and cultured for 24 h and then treated with fraction II in different concentration (0, 50, and 150 *μ*g/mL) for 48 h. The experiments was performed according to the manufacturer's instructions of kit; HepG2 cells were digested with trypsin (non-EDTA) and collected after being washed by PBS twice. Afterwards, the cells were suspended in 500 *μ*l binding buffer then stained with 5 *μ*l Annexin V-FITC and 5 *μ*l propidium iodide (PI) for 15 min at room temperature in the dark. The samples were immediately detected using BD FACS Canto II flow cytometer.

### 2.7. Data Analysis

Data were expressed as mean as the mean ± SEM and the results from each group were obtained using triplicate samples independently. One-way analysis of variance (ANOVA) and Fisher's least significant difference (LSD) test were used to determine significant differences for different samples. A probability value of ^*∗*^ *P* < 0.05 was considered to be statistically significant while ^*∗∗*^ *P* < 0.01 was considered to be highly statistically significant and are indicated in the figures. All statistical analysis were conducted using SPSS 20.0 and Prism 5 software package (GraphPad Software Inc., La Jolla, CA, USA).

## 3. Results

**Figure 1:**
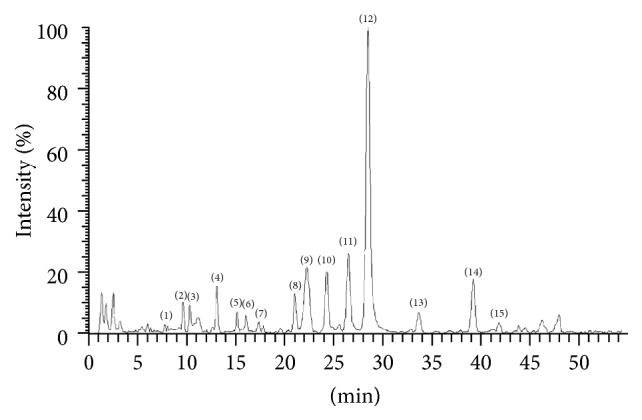
The UHPLC-ESI/MS (TIC) fingerprint of* Dendrobium aurantiacum* var. denneaum. Apigenin-6,8-di-C-*β*-*D*-glucoside (3), Apigenin-6-c-*β*-D-xyloside-8-C-*β*-D-glucoside (4), Isoschaftoside (5), Schaftoside (6), Quercetin-3-O-rutinoside-7-O-glucoside (7), Apigenin-6-C-*β*-D-glucoside-8-c-*β*-*D*-xyloside (8), Apigenin-6-C-glucosyl-2′′-O-xyloside (9), Apigenin-8-C-glucosyl-2′′-O-xyloside (10), Quercetin-7-O-rutinoside (11), Rutin (12), Kaempferol-3-O-rutinoside (13), Kaempferol-7-O-rutinoside (14), Apigenin-7-O-rutinoside (15).

Take adequate amounts of the four fractions for HPLC test. Most of the flavones were in fraction II while very few of them can be detected in fraction III. No flavones can be spotted in fractions I and IV. And, as a result, the fraction II was applied for further experiments including qualitative analysis of the flavonoids and cell experiments. From Figure 1 we can see that, at the wavelength of 340 nm, 15 chromatographic peaks can be found in fraction II. In order to clarify the structure of these components, UHPLC-ESI-MS/MS was applied.

### 3.1. Identification of Flavonoid Glycosides

Comparing the RT (retention time) of UV and ESI-MS^*n*^ spectra with literature data, we speculated and identified the potential chemical structures of 13 main essential ingredients (expect peaks (1) and (2) which were not detected), including flavonoid-O-glycosides and flavonoid-C-glycosides. The RT, MS, and MS^2^ spectral information and the identification results of the flavonoids were listed in Table 1.

**Figure 2:**
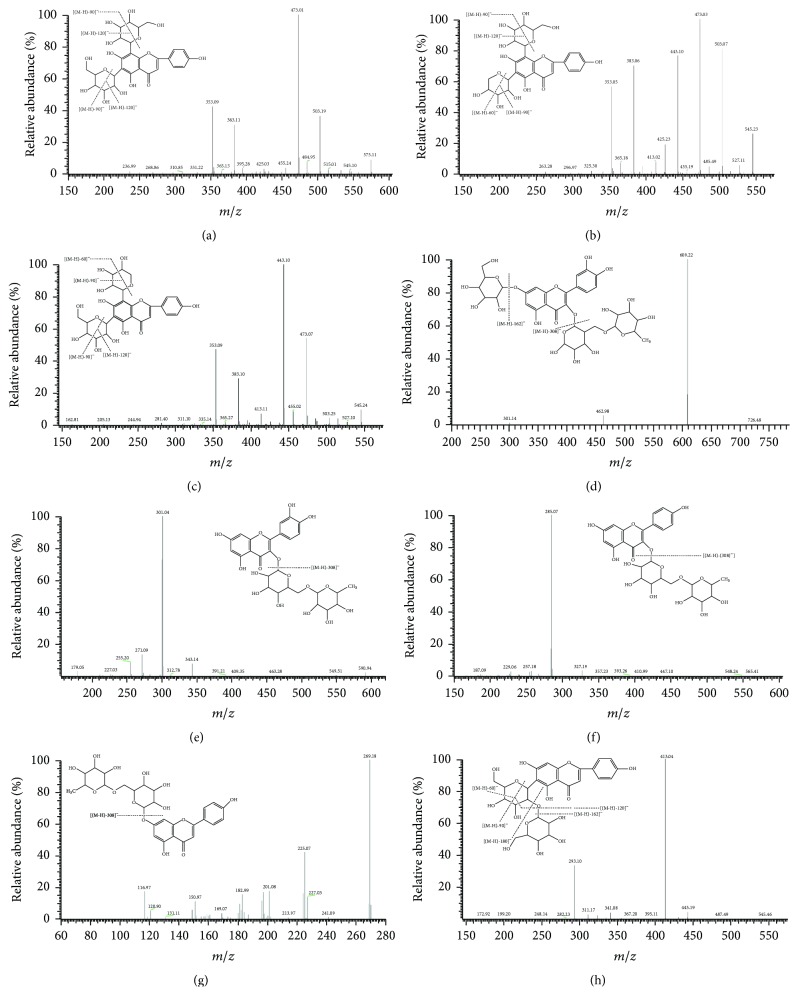
Chemical structures and MS/MS spectra of 8 flavonoids, Apigenin-6,8-di-C-*β*-glucoside (a), Isoschaftoside (b), Schaftoside (c), Quercetin-3-O-rutinoside-7-O-glucoside (d), Rutin (e), Kaempferol-3-O-rutinoside (f), Apigenin-7-O-rutinoside (g), Apigenin-6-C-glucosyl-2′′-O-xyloside (h).

In the present study, 5 flavonoid-c-glycosides have been identified in the* Dendrobium aurantiacum* var. denneaum including peaks (3), (4), (5), (6), and (8). A series of characteristic fragment patterns of flavonoid-c-glycosides arise from the fracture of the glucosyl as follows: the major fragmentation pathways were parent fragment ions loss of [(M-H)-60]^–^, [(M-H)-90]^–^ and [(M-H)-120]^–^, [(M-H)-90-120]^–^, [M-H-2 × 120]^–^, [(M-H)-120-CO]^–^, [(M-H)-2 × 120-2CO]^–^, and so on in MS or MS/MS. The glycosides of flavonoids-C-glycosides in* D. denneaum paxt.* are usually replaced in the C-6 and C-8 position. Furthermore, the glycosides on the C-6 position revealed more commonly ion fragmentation than C-8 position. [M+H]^+^ and [M+Na]^+^ ions were the primary positive ESI/MS in* D. denneaum paxt.*, in which further fragment was [M+H-18]^+^ resulting from continuous losses of one molecule of H_2_O. The characteristic fragment indicated that the positive negative ion mode could not provide much help to structure determination. Peak (3) showed [M-H]^–^ at *m*/*z* 593, the fragment ion peak showed in Figure 2(a), including 473 [(M-H)-120]^–^ and 353 [(M-H)-120-90]^–^; it was identified as Apigenin-6,8-di-C-*β*-*D*-glucoside by comparing with the standard. Peaks (4) and (5) had a molecular ion [M-H]^–^ at *m*/*z* 563, which produced similar MS^2^ major fragments at *m*/*z* 473 [(M-H)-90]^–^ and MS^3^ at *m*/*z* 353 [(M-H0-90-120]^–^, as shown in Figure 2(b); upon comparison with standards, peaks (4) and (5) were established as Apigenin-6-C-*β*-*D*-xyloside-8-C-*β*-*D*-glucoside and Isoschaftoside. Peaks (6) and (8) (in Figure 2(c)) also showed the same molecular ion [M-H]^–^ at *m*/*z* 563, yielding produced fragments at *m*/*z* 443 [(M-H)-120]^–^ and 353 [(M-H)-120-90]^–^. Meanwhile, accompanied with ions at *m*/*z* 503 [(M-H)-60]^–^, *m*/*z* 383 [(M-H)-120-60]^–^ and *m*/*z* 353 [(M-H)-120-90]^–^ have also been found. Moreover, these findings are consistent with the literature data. Compounds 6 and 8 were unambiguously identified as Schaftoside and Apigenin-6,8-di-C-*α*-*L*-arabinoside by comparing with the standard.

Peaks (7), (11), (12), (13), (14), and (15) showed similar fragmentation behaviours with the loss of 308, 176, 162, and 146 mass units showing the potential existence of flavonoid-o-glycosides, while 308 Da was proved as the typical fragment of Rutinose. The glycosides ordinarily took place on the position of C-3 and C-7; the relative intensity of [(M-2H)-gly]^–^ higher than [(M-H)-gly]^–^ indicated that the glycosides superseded on the C-3 position while the C-7 position is completely the opposite. For instance, Rutin, [M-H]^–^ ion, at *m*/*z* 300 possessed higher intensity than *m*/*z* 301. Peaks (7), (12), and (13) were assigned as 3-O-rutinosides, whereas peaks (11), (14), and (15) were assigned as 7-O-Rutinosides. Peak (7) showed [M-H]^–^ ion at *m*/*z* 771, and further fragmentation at *m*/*z* 609 ([M-H]-162)^–^ (Figure 2(d)), 300 ([M-H]-162-308)^–^, and 301 ([M-2H]-162-308)^–^, suggesting the presence of Rutinose and Quercetin in the structure; it is tentatively identified as Quercetin-3-O-rutinoside-7-O-glucoside. The parent ions at 609.34 [M-H]^–^ and 610.82 [M+H]^+^ of peak (11) were similar to that of peak (12). It means that molecular mass of peaks (11) and (12) was 610, which produced fragments at *m*/*z* 301 [(M-H)-308]^–^, *m*/*z* 300 [(M-2H)-308]^–^. The *m*/*z* 303 that appeared in positive ion mode of peaks (11) and (12) was the proof that Quercetin existed. Peak (11) was tentatively determined as Quercetin-7-O-rutinoside. Peak (12) (showed in Figure 2(e)) was unambiguously identified as Rutin through comparison with the standard compound. Peaks (13) and (14) shared ions of [M-H]^–^ at *m*/*z* 593 and exhibited fragments at *m*/*z* 285, *m*/*z* 284, *m*/*z* 163, and *m*/*z* 151; *m*/*z* 285 and *m*/*z* 284 indicated that these compounds contain Kaempferol. Based on the chromatographic column behaviour, peak (13) was assigned as Kaempferol-3-O-rutinoside; compound (14) was identified as Kaempferol-7-O-rutinoside (Figure 2(f)). Peak (15) had a [M-H]^–^ ion at *m*/*z* 577, which produced second-level fragment at *m*/*z* 269.06 (confirmed as Apigenin). Thus, compound (15) was deduced as Apigenin-7-O-rutinoside (Figure 2(g)); it has been reported in reference articles.

Peaks (9) and (10) showed the same [M-H]^–^ ion at *m*/*z* 563.13, which generated the peaks at *m*/*z* 413 [M-H-150]^–^, *m*/*z* 443 [M-H-120]^–^, and *m*/*z* 293 [M-H-120-150]^–^; this study led to the identification of C-glucosyl-2′′-O-xyloside. Compound (9) was assigned as Apigenin-6-C-glucosyl-2′′-O-xyloside and compound (10) was identified as Apigenin-8-C-glucosyl-2′′-Oxyloside. Both of them were initially identified through comparing their mass spectrum and UV spectral values, which have been identified in Citrus fruit juices. The results were shown in Figure 2(h).

### 3.2. Total Flavonoids Content

The total content of flavonoids components enriched fraction from* D. denneaum paxt. *was 1792.11 ug RE/g.

### 3.3. Antitumor Activity of Fraction II

#### 3.3.1. Antiproliferative Effect of Fraction II in HepG2 Cell

**Figure 3:**
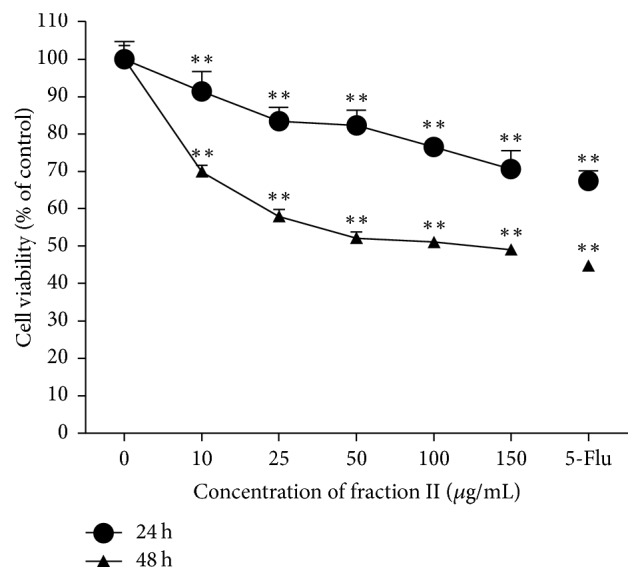
Effect of fraction II on the inhibition of HepG2 cell growth. Measurement of the effect of fraction II on the cytotoxicity in HepG2 cells using MTT assay. The vertical axis showed the cell viability culture with different corresponding concentration of the fraction II on horizontal axis. The lines of ● and ▲ represented incubation time of 24 h and 48 h. The cell viability was decreased dose- and time-dependently following incubation with 10, 25, 50, 100, and 150 *μ*g/mL fraction II for 24 h and 48 h. The concentration of 5-Flu was 50 *μ*g/mL as the positive drug group. ^*∗∗*^ *P* < 0.01 compared to the control group (0 *μ*M fraction II).

The antitumor effect of fraction II on human liver cancer cell HepG2 was measured by MTT assay. Different concentrations of fraction II were used to investigate the effects on the cell viability of HepG2. As it is shown in Figure 3, fraction II inhibited the growth of HepG2 cells in a dose-dependent and time-dependent manner after being induced for 24 h and 48 h. When comparing fraction II treatment group with the control group and positive drug group (5-Flu), the appropriate duration time of fraction II treatment was 48 h. After being treated for 48 h, the cell viability will decrease to 50.91%. Interestingly, even though the treatment concentration level reaches over 50 *μ*g/mL, the cell viabilities will not decrease markedly and are only maintained at a level of about 50% in 48 h. According to the statistical analysis, there was no statistical significance between the cell viability of the treatment groups in three different concentration levels at 50, 100, and 150 *μ*g/mL after 48 h. Therefore, we would like to use the concentration levels at 50 *μ*g/mL and 150 *μ*g/mL to proceed with further experiments to find out whether the morphological changes and apoptosis rate of the HepG2 cells would be the same like the MTT assay results.

#### 3.3.2. Anticancer Effect of Fraction II on the Morphological Changes of HepG2 Cell

**Figure 4:**
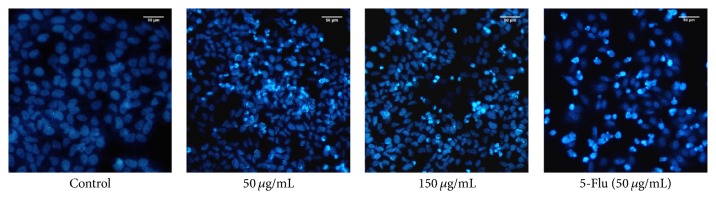
Effect of fraction II on the morphological changes in HepG2 cells using fluorescence microscopy. Nuclear staining with Hoechst 33258 using fluorescence microscopy showed that fraction II induces typical cell apoptosis including membrane blebbing, cytoplasm shrinkage, nuclear chromatin condensation, and nucleoli fragmentation.

The apoptotic morphology and morphological changes of HepG2 cells were observed by fluorescence microscopy. As it is shown in Figure 4, after the treatment with fraction (50 and 150 *μ*g/mL) for 48 h, the number of cells decreased and typical characteristics of apoptosis phenomenon such as membrane blebbing, cytoplasm shrinkage, nuclear chromatin condensation, and nucleoli fragmentation of cells can be observed. However, there were no significant differences between the cells treated with 50 and 150 *μ*g/mL which is similar to MTT assay. That is to say fraction II can effectively inhibit the growth of human liver cancer HepG2 cells by inducing apoptosis.

#### 3.3.3. Anticancer Effect of Fraction II on the Apoptosis Rate of HepG2 Cells

**Figure 5:**
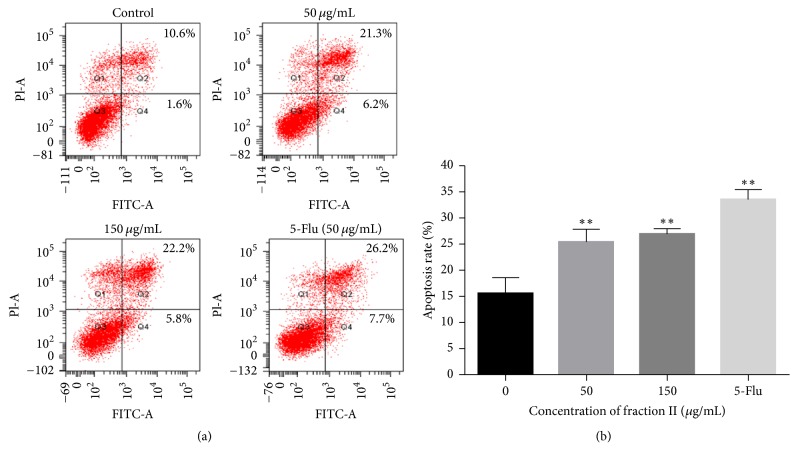
(a) Effect of fraction II on cell apoptosis in HepG2 cells. (b) HepG2 cells were treated with 0, 50, and 150 *μ*g/mL of fraction II for 48 h and the percentage of apoptotic cells was determined using Annexin V-FITC and PI. The concentration of 5-Flu was 50 *μ*g/mL as the positive drug group. ^*∗∗*^ *P* < 0.01 compared with the control group (0 *μ*M fraction II).

In order to explore the apoptosis rate of HepG2, we applied the Annexin V and PI double-staining assay kit to study using flow cytometry. As it is shown in Figure 5(b), after incubation with fraction II at the concentration of 50 and 150 *μ*g/mL for 48 h, the apoptosis rates were 25.4% and 27.0%, respectively. Although there were no significant differences between the cells induced with 50 and 150 *μ*g/mL fraction II, they were both remarkably higher than the control group, and almost reaching the apoptosis rate of the positive drug group, which exhibited a significant increase in the cell apoptosis rate. This result was also similar to MTT assay and demonstrates once again that fraction II has an effectively apoptotic effect on HepG2 cell when the concentration was over 50 *μ*g/mL for 48 h.

## 4. Discussion

Flavonoid is a group of phenols with low molecular weight which widely exists in plants. Some of them have been proved to exhibit certain antitumor activities, and the mechanism was clarified by researchers [24].* Dendrobium* genus is the largest in genus in Orchid and they have been used for thousand years in the mainland of China, as it was recorded that they exhibit certain pharmacology function such as nourishing the stomach and enhancing production of body fluids [25]. In the modern pharmacology study, researchers started to recognize the high value plants from* Dendrobium *genus that exhibit high efficacy on antitumor activities, such as* Dendrobium officinale*. Fan and Luo studied the antitumor activity of the polysaccharides from* Dendrobium denneaum* and found that the purified polysaccharides fractions DDP1-1 exhibit strong antitumor activities [26]. Haizhen et al. studied the antitumor activities of* Dendrobium officinale* polysaccharides and found that polysaccharides fraction DOPA can inhibit the growth of MCF-7 cell line. And the mechanism may be related to the PI3 K/AKT pathway [27]. However, most studies of the antitumor activities of* Dendrobium* genus mainly focus on the polysaccharides or crude extract, not the purified flavonoids fractions. So, in this study, we investigated the antitumor activities of the flavonoids from* D. denneaum paxt. *and found that the flavonoids' fraction II exhibits strong antitumor activities on human liver cancer cells HepG2. Furthermore, we preliminarily confirmed that the antitumor activities effects were through inducing apoptosis of HepG2 cells and the effective concentration and duration time was at a concentration above 50 *μ*g/mL for 48 h of the flavonoids from* D. denneaum paxt.*

We applied separation technology to obtain flavonoids fractions from* D. denneaum paxt.* and tested their antitumor activities. AB-8 macroporous resin was used to enrich and purify the crude extract. After being eluted with ethanol in different concentration, four fractions were collected and most of flavones were in fraction II. The results of UHPLC-MS showed that there were 15 chromatographic peaks in the flavones of the* D. denneaum paxt.* And the structure of 13 peaks was elucidated. When comparing the characterized peaks to the former studies published previously by Ye et al. [28], some compounds such as two Apigenin glucosides, Isoschaftoside, Schaftoside, and Rutin identified in this study were also identified by the previous paper. In this study, four Apigenin glucosides, two Kaempferol glucosides, and two Quercetin were identified from* D. denneaum paxt. *for the first time. Apart from the qualitative analysis of flavonoids, the total flavonoids content was also tested. There is a substantial content of flavones constituents in* D. denneaum paxt.*; the content of the Rutin especially is exceptionally high, which possesses many potential bioactive properties.

## 5. Conclusion

The study of natural plants products is becoming more and more popular all around the world. As the human society is developing faster and faster, the tendency of population to age would raise more attention to the medical care issue. In this study we proved that the flavonoids in* D. denneaum paxt. *exhibit certain antitumor and proapoptotic activities. So this plant has the potential to be developed into a pharmaceutical and nutraceutical products.

## Figures and Tables

**Table 1:** MS date for characterization of compounds in *Dendrobium denneaum paxt.* by UHPLC-ESI-MS/MS.

Peak number	RT (min)	RRT	Negative ions (*m*/*z*)	MS^n^	Identification
(3)	9.5	0.24	593 [M-H]^–^	MS^2^: 503.19, 473.01 (100), 383.11, 353.09	Apigenin-6,8-di-c-*β*-D-glucoside
(4)	11.98	0.28	563 [M-H]^–^	MS^2^: 503.08, 473.04 (100), 443.08, 383.10, 353.07	Apigenin-6-C-*β*-D-xyloside-8-C-*β*-D-glucoside
(5)	14.53	0.26	563 [M-H]^–^	MS^2^: 503.07, 473.03 (100), 443.10, 383.06, 353.05	Isoschaftoside
(6)	15.2	0.17	563 [M-H]^–^	MS^2^: 473.07, 443.10 (100), 383.10, 353.09	Schaftoside
(7)	17.58	0.09	771 [M-H]^–^	MS^2^: 609.22 (100), 462.98; MS^3^: 301.06	Quercetin-3-O-rutinoside-7-O-glucoside
(8)	19.57	0.26	563 [M-H]^–^	MS^2^: 473.09, 443.10 (100), 383.18, 353.12	Apigenin-6-C-*β*-D-glucoside-8-C-*β*-D-xyloside
(9)	21.6	1	563 [M-H]^–^	MS^2^: 413.04 (100), 293.06; MS^3^: 293.08	Apigenin-6-C-glucosyl-2′′-O-xyloside
(10)	24.05	1.09	563 [M-H]^–^	MS^2^: 413.04 (100), 293.10 MS^3^: 293.09	Apigenin-8-C-glucosyl-2′′-O-xyloside
(11)	25.95	0.5	609 [M-H]^–^	MS^2^: 300.02 (100), 271.06 MS^3^: 271.03, 255.05, 179.00, 150.94	Quercetin-7-O-rutinoside
(12)	28.05	2.02	609 [M-H]^–^	MS^2^: 301.04 (100), 271.09; MS^3^: 271.07, 255.08, 179.00, 151.01	Rutin
(13)	33.13	0.13	593 [M-H]^–^	MS^2^: 285.0 MS^3^: 267.06, 257.06 (100), 225.16, 229.06, 212.99	Kaempferol-3-O-rutinoside
(14)	38.86	0.39	593 [M-H]^–^	MS^2^: 285.07 MS^3^: 267.19, 257.13 (100), 229.01, 213.18	Kaempferol-7-O-rutinoside
(15)	42.05	0.13	577 [M-H]^–^	MS^2^: 269.18 (100), 225.07	Apigenin-7-O-rutinoside

## Abbreviations

*D. denneaum paxt.*: * Dendrobium denneaum paxt.*
TCM: Traditional Chinese medicine
UHPLC-ESI-MS/MS: Ultra-high performance liquid chromatography-electrospray ionization/mass spectrometry
RT: Retention times.

## References

[1] Chen N.-D., Chen N.-F., Li J., Cao C.-Y., Wang J.-M., Huang H.-P. (2015). Similarity Evaluation of Different Origins and Species of Dendrobiums by GC-MS and FTIR Analysis of Polysaccharides. *International Journal of Analytical Chemistry*.

[2] Wu C., Gui S., Huang Y. (2016). Characteristic fingerprint analysis of: Dendrobium huoshanense by ultra-high performance liquid chromatography-electrospray ionization-tandem mass spectrometry. *Analytical Methods*.

[3] Tong L., Wang L., Zhou X. (2016). Antitumor activity of Dendrobium devonianum polysaccharides based on their immunomodulatory effects in S180 tumor-bearing mice. *RSC Advances*.

[4] Xing Y.-M., Chen J., Cui J.-L., Chen X.-M., Guo S.-X. (2011). Antimicrobial activity and biodiversity of endophytic fungi in Dendrobium devonianum and Dendrobium thyrsiflorum from Vietman. *Current Microbiology*.

[5] Taheri S., Abdullah T., Karimi E., Oskoueian E., Ebrahimi M. (2014). Antioxidant Capacities and Total Phenolic Contents Enhancement with Acute Gamma Irradiation in Curcuma alismatifolia (Zingiberaceae) Leaves. *International Journal of Molecular Sciences*.

[6] Zhang J., He C., Wu K. (2016). Transcriptome analysis of dendrobium officinale and its application to the identification of genes associated with polysaccharide synthesis. *Frontiers in Plant Science*.

[7] Pan L.-H., Li X.-F., Wang M.-N. (2014). Comparison of hypoglycemic and antioxidative effects of polysaccharides from four different *Dendrobium species*. *International Journal of Biological Macromolecules*.

[8] Huang X., Nie S., Cai H. (2015). Study on *Dendrobium officinale* O-acetyl-glucomannan (Dendronan®): part VI. Protective effects against oxidative stress in immunosuppressed mice. *Food Research International*.

[9] Xu J., Li S.-L., Yue R.-Q. (2014). A novel and rapid HPGPC-based strategy for quality control of saccharide-dominant herbal materials: Dendrobium officinale, a case study. *Analytical and Bioanalytical Chemistry*.

[10] Wu H., Guo J., Chen S. (2013). Recent developments in qualitative and quantitative analysis of phytochemical constituents and their metabolites using liquid chromatography-mass spectrometry. *Journal of Pharmaceutical and Biomedical Analysis*.

[11] Kroslakova I., Pedrussio S., Wolfram E. (2016). Direct Coupling of HPTLC with MALDI-TOF MS for Qualitative Detection of Flavonoids on Phytochemical Fingerprints. *Phytochemical Analysis*.

[12] Cao J., Xia X., Chen X., Xiao J., Wang Q. (2013). Characterization of flavonoids from *Dryopteris erythrosora* and evaluation of their antioxidant, anticancer and acetylcholinesterase inhibition activities. *Food and Chemical Toxicology*.

[13] Mphahlele R. R., Stander M. A., Fawole O. A., Opara U. L. (2014). Effect of fruit maturity and growing location on the postharvest contents of flavonoids, phenolic acids, vitamin C and antioxidant activity of pomegranate juice (cv. Wonderful). *Scientia Horticulturae*.

[14] Terpinc P., Cigić B., Polak T., Hribar J., Požrl T. (2016). LC-MS analysis of phenolic compounds and antioxidant activity of buckwheat at different stages of malting. *Food Chemistry*.

[15] Oszmiański J., Wojdyło A., Nowicka P., Teleszko M., Cebulak T., Wolanin M. (2015). Determination of phenolic compounds and antioxidant activity in leaves from wild Rubus L. species. *Molecules*.

[16] Šibul F., Orčić D., Vasić M. (2016). Phenolic profile, antioxidant and anti-inflammatory potential of herb and root extracts of seven selected legumes. *Industrial Crops and Products*.

[17] Zhang X., Huang H., Zhang Q. (2015). Phytochemical characterization of Chinese bayberry (Myrica rubra Sieb. et Zucc.) of 17 cultivars and their antioxidant properties. *International Journal of Molecular Sciences*.

[18] Benayad Z., Gómez-Cordovés C. (2014). Characterization of flavonoid glycosides from fenugreek (Trigonella foenum-graecum) crude seeds by HPLC-DAD-ESI/MS analysis. *International Journal of Molecular Sciences*.

[19] Hu Q., Yu J., Yang W. (2016). Identification of flavonoids from Flammulina velutipes and its neuroprotective effect on pheochromocytoma-12 cells. *Food Chemistry*.

[20] Thong N. M., Dao D. Q., Ngo T. C., Huyen T. L., Nam P. C. (2016). Antioxidant activities of [60]fullerene derivatives from chalcone, flavone and flavanone: A ONIOM approach via H-atom and electron transfer mechanism. *Chemical Physics Letters*.

[21] Jiang Q., Li X., Tian Y. (2017). Lyophilized aqueous extracts of Mori Fructus and Mori Ramulus protect Mesenchymal stem cells from •OH-treated damage: Bioassay and antioxidant mechanism. *BMC Complementary and Alternative Medicine*.

[22] Yuan L., Wang J., Wu W., Liu Q., Liu X. (2016). Effect of isoorientin on intracellular antioxidant defence mechanisms in hepatoma and liver cell lines. *Biomedicine & Pharmacotherapy*.

[23] Zou Y., Chang S. K. C., Gu Y., Qian S. Y. (2011). Antioxidant activity and phenolic compositions of lentil (*Lens culinaris* var. Morton) extract and its fractions. *Journal of Agricultural and Food Chemistry*.

[24] Aydemir E. Flavonoids as anti-cancer agents and their mechanism of action.

[25] Cakova V., Bonte F., Lobstein A. (2017). Dendrobium: Sources of Active Ingredients to Treat Age-Related Pathologies. *Aging and Disease (A&D)*.

[26] Fan Y., Luo A. (2011). Evaluation of anti-tumor activity of water-soluble polysaccharides from Dendrobium denneanum. *African Journal of Pharmacy and Pharmacology*.

[27] Haizhen B., Shengrong S., Yimin Z., Si S., Zhongwei C. (2017). Inhibitory effect of Dendrobium officinale polysaccharide on growth of human breast cancer MCF-7 cells and the related mechanism. *Biomedical Research (India)*.

[28] Ye Z., Dai J.-R., Zhang C.-G. (2017). Chemical Differentiation of Dendrobium officinale and Dendrobium devonianum by Using HPLC Fingerprints, HPLC-ESI-MS, and HPTLC Analyses. *Evidence-Based Complementary and Alternative Medicine*.

